# Fundamental Understanding and Optimization Strategies for Dual-Ion Batteries: A Review

**DOI:** 10.1007/s40820-023-01086-6

**Published:** 2023-05-01

**Authors:** Chong Chen, Chun-Sing Lee, Yongbing Tang

**Affiliations:** 1 Advanced Energy Storage Technology Research Center, Shenzhen Institute of Advanced Technology, Chinese Academy of Sciences, Shenzhen, 518055 People’s Republic of China; 2 University of Chinese Academy of Sciences, Beijing, 100049 People’s Republic of China; 3 Center of Super-Diamond and Advanced Film (COSDAF), City University of Hong Kong, Kowloon, 999077 Hong Kong, SAR People’s Republic of China

**Keywords:** Dual-ion batteries, Reaction mechanisms, Optimization strategies, Energy storage

## Abstract

There has been increasing demand for high-energy density and long-cycle life rechargeable batteries to satisfy the ever-growing requirements for next-generation energy storage systems. Among all available candidates, dual-ion batteries (DIBs) have drawn tremendous attention in the past few years from both academic and industrial battery communities because of their fascinating advantages of high working voltage, excellent safety, and environmental friendliness. However, the dynamic imbalance between the electrodes and the mismatch of traditional electrolyte systems remain elusive. To fully employ the advantages of DIBs, the overall optimization of anode materials, cathode materials, and compatible electrolyte systems is urgently needed. Here, we review the development history and the reaction mechanisms involved in DIBs. Afterward, the optimization strategies toward DIB materials and electrolytes are highlighted. In addition, their energy-related applications are also provided. Lastly, the research challenges and possible development directions of DIBs are outlined.

## Highlights

- The development history and the reaction mechanisms involved in dual-ion batteries (DIBs) are reviewed.
- The optimization strategies toward DIB electrodes and electrolytes and their energy-related applications are highlighted.
- The research challenges and possible development directions of DIBs are outlined.

## Graphical abstract

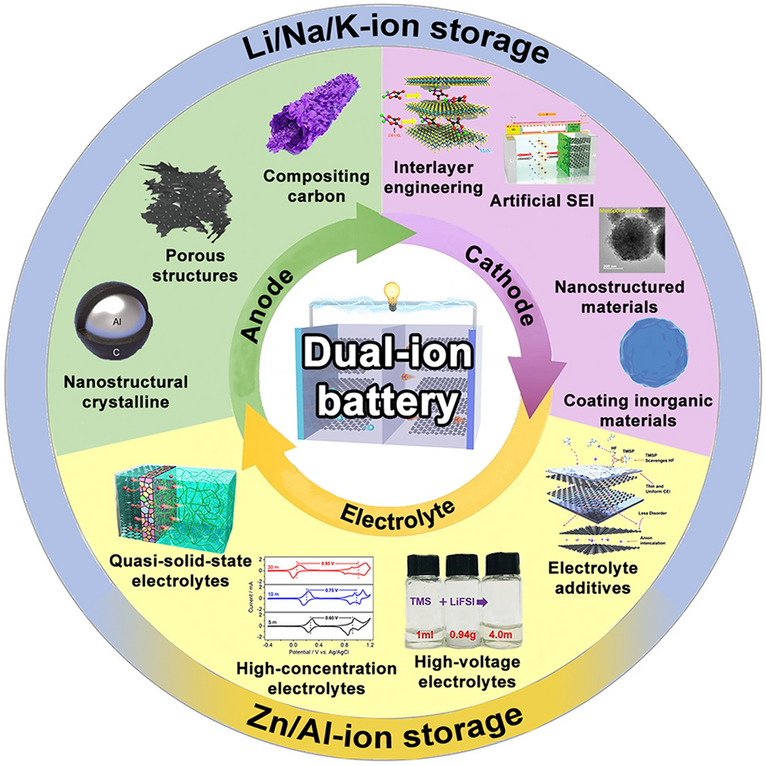

## Introduction

The increasing global energy demand and environmental issues are calling for the urgent development of efficient, sustainable, and carbon–neutral energy conversion and storage technologies [1–6]. In the past few years, lithium-ion batteries (LIBs) have attracted extensive attention due to their merits of high energy density and good cycling stability [7–12]. As LIBs expand their territory from consumer electronics to electric vehicles, a great concern arises about the widespread availability and rising price of lithium resources. Therefore, the exploration of low-cost rechargeable battery systems with high performance is of great importance to meet the rigid requirements for commercialization [13–15]. Among them, dual-ion batteries (DIBs) have been regarded as one of the most appealing alternatives to LIBs with intriguing features of high operating voltage, fast intercalation kinetics, and cost-efficiency [16–20]. At present, most advanced commercial LIBs are based on nickel (Ni) and cobalt (Co)-containing cathodes, generating cost rise and geopolitical tensions. Moreover, their recycling process is also difficult and environmentally unfriendly. The lead-acid batteries using Pb and PbO_2_ electrodes show substantial advantages such as low cost, stable voltage profile, and good safety. However, they still face low energy density and limited cycle life due to the grid corrosion and hydrogen evolution. Since carbonaceous materials such as graphite can be utilized as the cathode, DIBs display obvious advantages of low price and natural abundance compared to traditional battery systems. Table 1 presents the electrochemical properties and cost estimation of these commercial electrochemical battery systems. With the addition of their low cost and environmental benignancy, DIB is emerging as a significant type of energy storage device in the post-LIBs era. Despite a similar energy storage mechanism at the anode side to the traditional “rocking-chair” batteries like LIBs, DIBs typically feature intercalation of anions at the cathode materials. Furthermore, the electrolyte in DIBs can serve as both the ion transport medium and the active material. As a result, the DIBs are favorable for increasing energy density and meeting the requirements of commercial applications [21, 22].

**Table 1:** Summary of various energy storage systems and their electrochemical performance (the number of asterisks in the Table 1 represents the environment friendless level)

Batteries	Mass energy density (Wh kg^−1^)	Cost (USD/kWh)	Working voltage (V)	Environmental friendless
Lead-acid	30–70	67	2	***
LIBs	100–160	97	3.3–3.7	****
Al||graphene DIB	100–200	70	4.0–4.5	*****
Graphene||graphene DIB	100–180	40	4.0–5.0	*****

The first reversible intercalation phenomenon of HSO_4_^−^ into graphite was found by Rüdorff and Hofmann in 1938 [23]. They developed a cell with two graphite electrodes and concentrated H_2_SO_4_ as the electrolyte. In the 1990s, McCullough et al. [24] proposed a “dual-intercalation” mechanism in their patents, and later realized by Carlin et al. [25] with graphite as both the cathode and anode, and molten salt as the electrolyte in 1994. Afterward, the studies in this field mostly focused on the intercalation mechanisms of various anions or cations, as well as the electrolyte based on different solvents [26-30]. Among these studies, Seel et al. [31] realized the reversible PF_6_^−^ intercalation at the graphite cathode in 2000, and in 2014, Placke et al. [32] reported the stable cycling of DIBs based on the ionic liquid electrolyte. In 2016, Tang et al. optimized the cell configuration of DIBs by adopting Al foil as both the anode materials and current collector, which significantly reduced the cost and enhanced the energy density of DIBs [33]. Recently, Wang et al. synthesized a composite electrode containing equimolar lithium halide salts (LiBr)_0.5_(LiCl)_0.5_-graphite [34]. By employing highly concentrated water-in-bisalt electrolyte, this composite realized the anionic-redox reaction of halide anions (Br^−^ and Cl^−^) reversibly intercalation into the graphite host as solid graphite intercalation compounds. Thereafter, DIBs have been applied to various resourceful alkaline metals (Na, K) and even multivalent alkaline metals like Ca, Zn, Mg, and Al [35–38]. Although the rapid development of DIBs has been achieved, the related research is still at their early stage. More efforts should be devoted to exploring novel electrode materials and electrolytes tailored for high-performance DIBs [37, 39–43]. Besides, effective strategies and characterization techniques are needed to deeply understand the fundamental working mechanisms of proposed DIBs. There already exist several review articles providing some information on DIBs, many of which focus on the cathode and anode materials only, while the discussions on the optimization strategies for DIBs are quite limited [44–46]. In this review, we begin with a brief introduction to the development history of DIBs and their reaction mechanism. Then, we highlight various optimization strategies and efforts toward high-performance DIBs systems and their practical applications. Finally, some brief conclusions and perspectives on the development of DIBs are provided to inspire more innovative research on this novel energy storage device.

## Reaction Mechanisms for DIBs

**Fig. 1:**
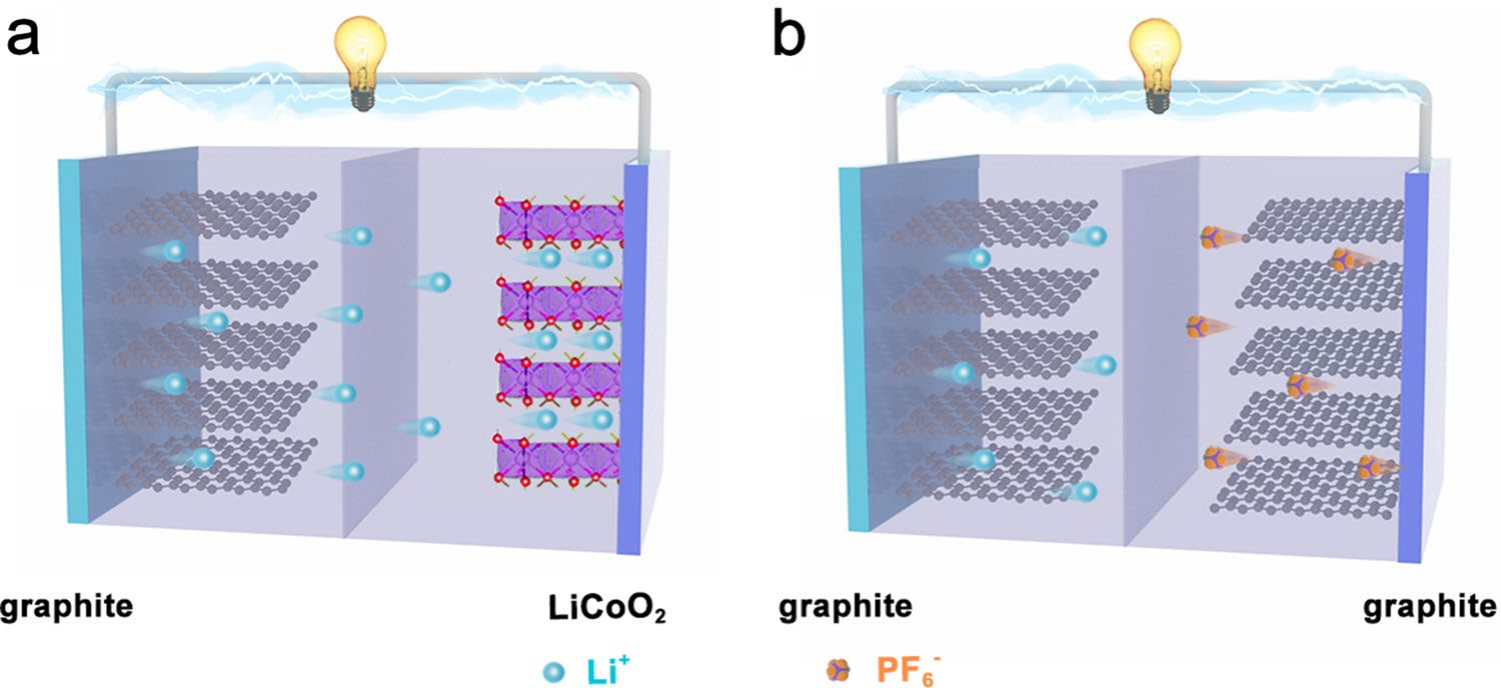
Schematic diagrams illustrating **a** a traditional LIB and **b** a dual-graphite DIB

Of particular note of the anion intercalation into the cathode during charge, the reaction mechanism of DIBs differs significantly from conventional LIBs [47, 48]. Figure 1a illustrates the charge–discharge mechanism of a traditional LIB using graphite as the anode and LiCoO_2_ as the cathode. Only Li^+^ reversibly shuttles between two electrodes, which is described as the “rocking chair” model [49]. Specifically, during the charging process, the Li^+^ de-insert from the LiCoO_2_ and intercalate into the graphite interlayers through the electrolyte. While for the discharge process, the Li^+^ de-insert from the graphite anode and intercalate into the LiCoO_2_ cathode. The migrated Li^+^ are generally provided by the cathode materials, and the electrolyte does not participate in the actual electrochemical energy storage process [50]. The charge reaction could be concluded as follows:

1 $${\text{Anode:}}\;x{\text{Li}}^{ + } + x{\text{e}}^{ - } + {\text{ C }} = {\text{Li}}_{x} {\text{C}}$$

2 $${\text{Cathode:}}{\text{ LiCoO}}_{{2}} = {\text{ Li}}_{{{1} - x}} {\text{CoO}}_{{2}} + x{\text{Li}}^{ + } + x{\text{e}}^{ - }$$

3 $${\text{Overall cell reaction:}}{\text{ LiCoO}}_{{2}} + {\text{ C }} = {\text{ Li}}_{{{1} - x}} {\text{CoO}}_{{2}} + {\text{ Li}}_{x} {\text{C}}$$

However, for the DIBs, both anion and cation from the electrolyte participate in the charge storage reaction (Fig. 1b). Take the dual-graphite DIB as an example, during the charging process, the cation (e.g., Li^+^) and anion (e.g., PF_6_^−^) are simultaneously intercalated in the dual-graphite anode and cathode. During the discharge process, the intercalated Li^+^ and PF_6_^−^ de-insert from the graphite and diffuse back to the electrolyte [51]. The charge reactions of the DIB based on graphite electrodes could be concluded as follows:

4 $${\text{Anode:}}\;x{\text{Li}}^{ + } + x{\text{e}}^{ - } + {\text{ C }} = {\text{Li}}_{x} {\text{C}}$$

5 $${\text{Cathode:}}{\text{ C }} + xA^{ - } = A_{x} {\text{C }} + x{\text{e}}^{ - }$$

6 $${\text{Overall cell reaction:}}x{\text{Li}}^{ + } + xA^{ - } + {\text{ C }} + {\text{ C }} = {\text{ Li}}_{x} {\text{C }} + A_{x} {\text{C}}$$

where *A*^−^ stands for anion in the electrolyte. Generally, the intercalation potential of anions on the graphite cathode can be up to 4.5 V (*vs.* Li^+^/Li), which is favorable for relatively high energy density [52]. Furthermore, instead of a high-cost lithium-containing transition metal oxide cathode, utilizing graphite as the cathode can effectively reduce the overall cost, and decrease potential environmental pollution [53]. Overall, compared to that LIBs, it must be recognized that the current DIBs are still at their fancy stage, and more explorations are necessary to further gain a comprehensive understanding of the fundamental reaction mechanisms and optimize their electrochemical performance.

## Optimization Strategies for High-Performance DIBs

**Fig. 2:**
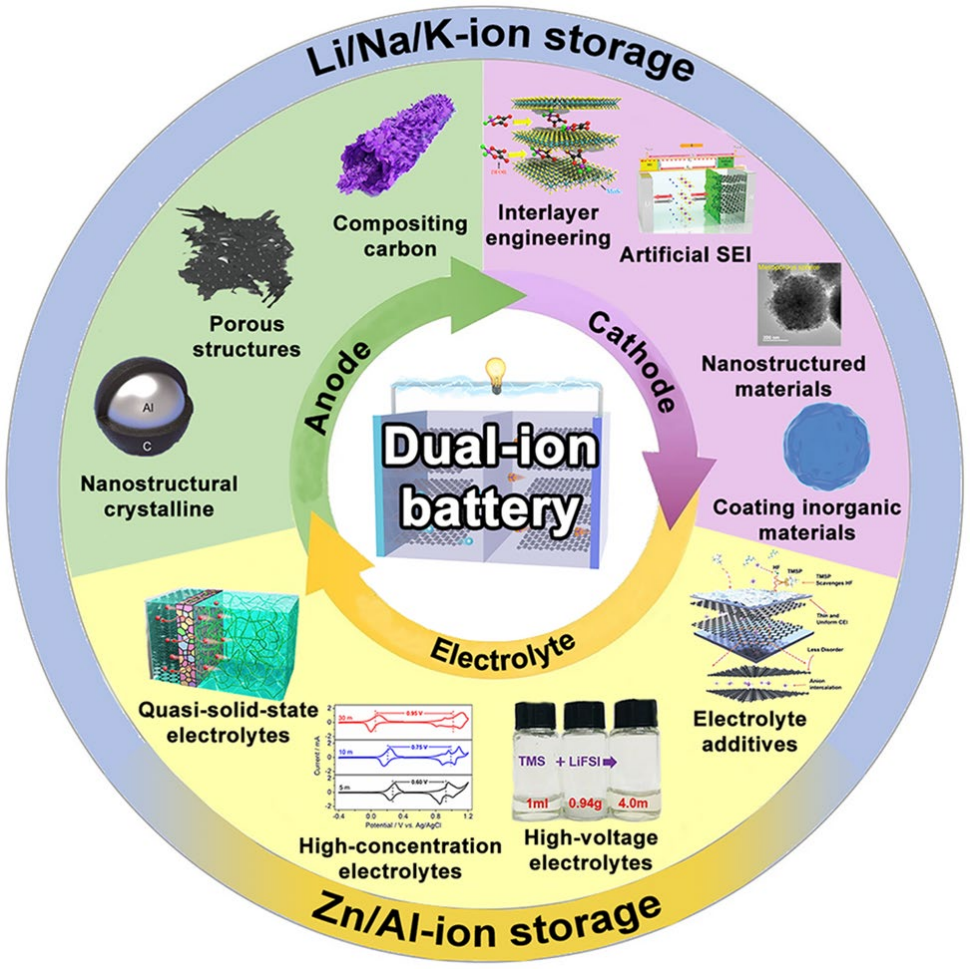
Summary of the emerging optimization strategies for DIBs covered in this review

Critical challenges are building efficient DIB configurations, such as relatively poor reaction kinetics of anode materials, unsatisfactory specific capacity with conventional cathode materials, and the mismatch of traditional electrolytes [54, 55]. To fully employ the advantages of DIBs, the overall optimization of anode materials, cathode materials, and compatible electrolyte systems is urgently needed to address the above issues (Fig. 2). Hence, in this section, we will discuss these optimization strategies in detail to find effective strategies for high-performance DIBs.

### Anode Optimization Strategies

**Fig. 3:**
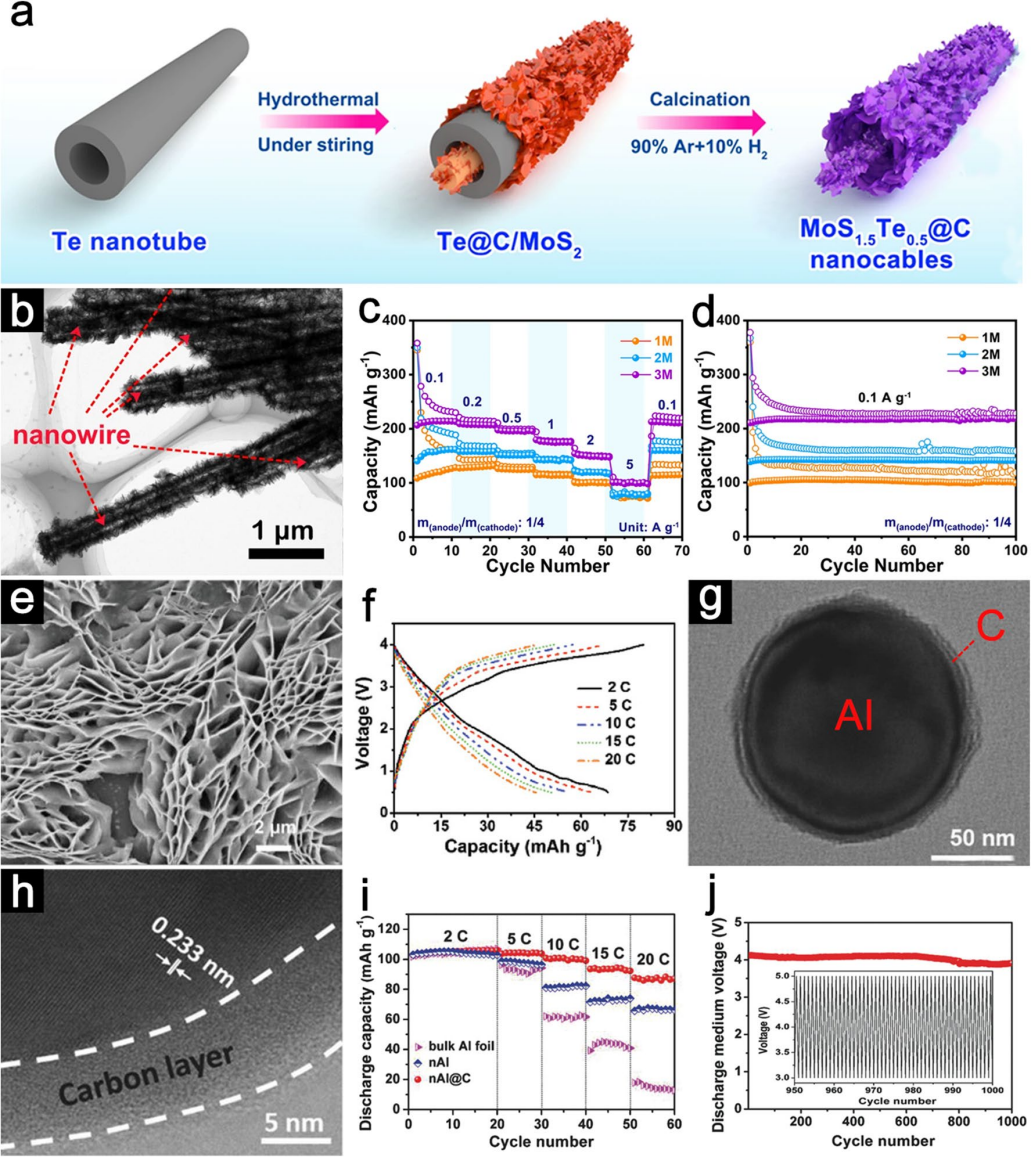
**a** Schematic illustration of the synthetic process for MoS_1.5_Te_0.5_@C nanocables. **b** TEM image of MoS_1.5_Te_0.5_@C nanocables. **c** Rate performance and **d** cycling stability of MoS_1.5_Te_0.5_@C nanocables||EG dual-ion cell. Panels (**a**–**d**) reproduced with permission from Ref. [61]. Copyright 2022, Nature Publishing Group. **e** SEM image of pK_2_TP nanosheets. **f** GCD curves of pK_2_TP||EG dual-ion cell. Panels (**e, f**) reproduced with permission from Ref. [66]. Copyright 2020, John Wiley & Sons, Inc. **g** TEM image and **h** HRTEM image of nAl@C nanosphere. **i** Rate capabilities and **j** cycling performance of nAl@C||G DIB. Panels (**g–j**) reproduced with permission from Ref. [70]. Copyright 2018, John Wiley & Sons, Inc

There are significant challenges facing the DIB anode materials, such as the limited specific capacity and their sluggish reaction kinetics, which relate to the mismatch with the cathode side, that need to be overcome [56, 57]. To address these issues, compositing active materials with highly conductive carbon, designing porous structures, and building nanostructural crystalline are reliable strategies for boosting the electrochemical performance of anode materials [39, 58, 59]. Combining anode materials with conductive carbon, such as graphene oxide, carbon nanotubes, and conducting polymers can be an effective strategy to enhance the electronic conductivity and increase the transfer rate of cations in the anode [60]. For example, Liu et al. developed a source-template approach for fabricating MoS_1.5_Te_0.5_@C nanocables with an in-situ grown carbon film coating (Fig. 3a) [61]. The outer surface of the one-dimensional structure is abundant with wrinkled nanosheets and the characteristic nanocable morphology can be observed (Fig. 3b). The carbonaceous film surrounding MoS_1.5_Te_0.5_ nanosheets acts as a protective layer to facilitate stable solid electrolyte interphase (SEI), which can decrease charge transfer resistance and enhance electrical conductivity. Owing to the structural and compositional advantages, the MoS_1.5_Te_0.5_@C composites demonstrate an enhanced Na-storage performance. A Na-DIB cell configuration is constructed using MoS_1.5_Te_0.5_@C as the anode and expand graphite (EG) as the cathode. Upon cycling in 3 M NaPF_6_-based non-aqueous electrolyte at current densities of 0.1, 0.2, 0.5, 1, 2, and 5 A g^−1^, the dual-ion cell delivers reversible capacities of 214.2, 207.8, 195.9, 175.8, 150.2 and 100.9 mAh g^−1^, respectively (Fig. 3c). Moreover, the MoS_1.5_Te_0.5_@C nanocables||EG dual-ion cell promises a high coulombic efficiency (CE) after 100 cycles (Fig. 3d). Placke et al. synthesized black phosphorus nanoparticles dispersed homogeneously in carbon (BP-C) of the composites as anode materials for DIB full-cell [62]. The active materials are confined in carbonaceous component, which could synergistically promote electronic conductivity and minimize the effects of significant volume changes of phosphorus. Based on LiTFSI in dimethyl carbonate (DMC) electrolyte, the BP-C||graphite DIB cell displays superior energy efficiency and cycling stability. Nevertheless, such strategies are sometimes accompanied with the compromise of intensified interlayer exfoliation, which are not satisfactory for achieving optimal electrochemical performance. To address this bottleneck, Yu et al. developed a strategy to selectively incorporate carboxylic anhydride functionality between graphite layers to stabilize the crystal structure [86]. Such configurations not only help to enlarge the interlayer distance which is beneficial for reversible ion insertion, but also prevent interlayer separation during cycling. Hence, the graphite shows enhanced stability and discharge capacity.

Designing anode materials with hierarchical porosity and structures is another common strategy for new energy storage and conversion systems [63–65]. The meticulous design and ingenious hierarchical structuration of porosities can provide good electrolyte infiltration, large surface areas for reaction, and improve ion diffusion kinetic at different length scales. By the incorporation of macroporosity in materials, their electrochemical performances can be greatly enhanced, showing the importance of microchannels in advanced energy storage materials. Yu et al. synthesized three-dimensional (3D) porous dipotassium terephthalate (pK_2_TP) nanosheets via a freeze-dry method as the K-DIB anode materials (Fig. 3e) [66]. The porous configuration possesses a higher ion diffusion coefficient and faster charge transfer kinetics compared with its bulk component. Benefiting from this novel multichannel structure and abundant active sites, a K-based DIB cell combining pK_2_TP as anode and EG as the cathode (pK_2_TP||EG), exhibits superior rate capability (up to 20C) and excellent cycling stability (2,000 cycles) (Fig. 3f). Wang et al. explored redox-active conjugated microporous polymers (RCMPs) by integrating copper (II) tetraaminephthalocyanine (CuTAPc) and 1,4,5,8-naphthalenetetracarboxylic dianhydride (NTCDA) units into the RCMPs (CuPcNA-CMP) [67]. This controllability of the porous structure offers fast electron/ion diffusion and reaction kinetics. As expected, the CuPcNA-CMP||graphite DIB shows a high reversible capacity (245.3 mAh g^−1^ at 0.1 A g^−1^) and good rate capability (125.1 mAh g^−1^ at 5 A g^−1^) in Li-based DIBs.

Designing electrode materials with nanoconfined architectures and controllable morphologies has many structure-dependent merits for electrochemical energy storage applications [68, 69]. The permeable nanosized building blocks and robust secondary frameworks can decrease the diffusion energy barrier for the migration of electrons/ions, and the large surface area endows accessible electroactive sites and good wettability for the electrolyte, thus can allow full utilization of the active materials and promote the reaction kinetics. The elaborate architecture with tailored size and composition helps to retain the integrity of the electrode to achieve long-term cycle life. Recently, Tong et al. fabricated core/shell aluminum@carbon (nAl@C) nanospheres as anode material for DIB (Fig. 3g) [70]. The resultant frameworks are composed of Al nanosphere as the inner core and ~ 5 nm thickness amorphous carbon as the outer layer (Fig. 3h). This unique nanoscale framework helps accommodate mechanical stress and inhibit Al pulverization. In addition, the conductive carbon layer is beneficial for conducting electrons and facilitating the formation of a stable SEI film during cycling, thus significantly enhanced the cycling stability of DIB. Owing to the delicate core–shell structural design, the nAl@C||graphite DIB demonstrates ultrahigh rate performance (88 mAh g^−1^ at 15C) as well as superior cycling stability (95.1% capacity retention after 1,000 cycles) (Fig. 3i-j). Salunkhe et al. reported the novel synthesis of Co_3_Sn_2_ and SnO_2_ core–shell heterostructures (Co_3_Sn_2_@SnO_2_) as anode materials for Li-based DIBs [71]. The Co_3_Sn_2_@SnO_2_||EG DIB cell delivers a reversible capacity of 90 mAh g^−1^ at 300 mAh g^−1^ and a high CE of 93.3%. In a recent study, Wu et al. provided an ingenious nanostructure engineering to fabricate *α*-Fe_2_O_3_ anode with a unique nano-cubic structure [39]. The *α*-Fe_2_O_3_ exhibits mesoporous structures, which can suppress the structural degradation and volume expansion caused by the conversion reaction. As the anode of a Li-based DIB, the battery displays a high discharge capacity (362 mAh g^−1^ at 0.5C) and rapid ion diffusion kinetics (5C for 520 cycles).

### Cathode Optimization Strategies

**Fig. 4:**
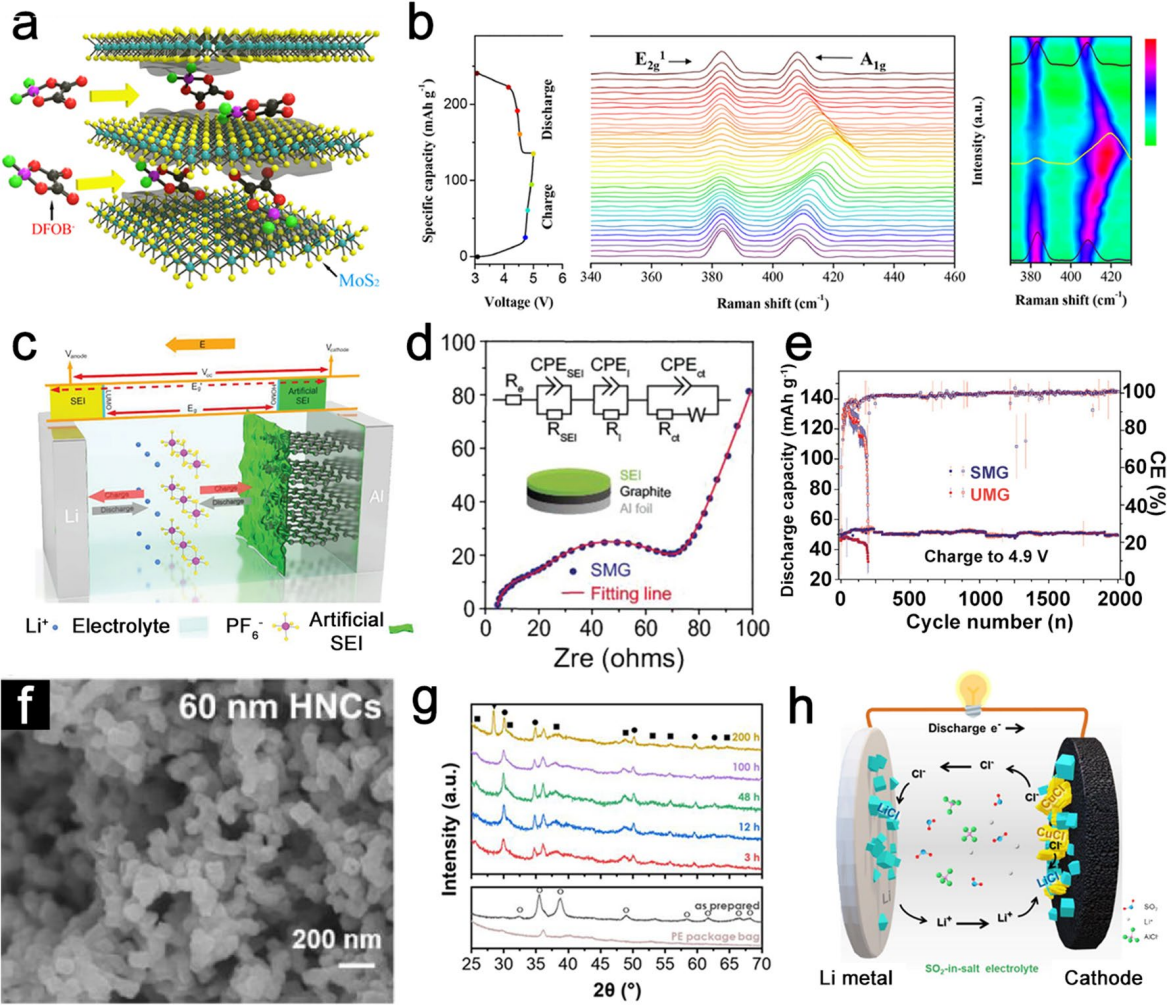
**a** Schematic illustration of MoS_2_ cathode with expanded interlayer. **b** In-situ Raman spectra of MoS_2_ cathode during charge/discharge process. Panels (**a, b**) reproduced with permission from Ref. [81]. Copyright 2018, John Wiley & Sons, Inc. **c** Schematic diagram illustration of the Li||SMG DIB. **d** Nyquist plot of the SMG electrode. The inset shows an equivalent circuit of the Li||SMG cell. **e** Cycling performance of SMG and UMG electrodes. Panels (**c–e**) reproduced with permission from Ref. [86]. Copyright 2020, Elsevier. **f** SEM image of CuO HNCs. **g** *Ex-situ* XRD patterns of CuO HNCs electrodes after 0, 3, 12, 48, 100, and 200 h immersion time in LiAlCl_4_⋅3SO_2_ electrolyte. **g** Schematic illustration of the Li||CuO HNCs DIB. Panels (**f–h**) reproduced with permission from Ref. [87]. Copyright 2021, Elsevier

In DIBs, anion intercalation is the most important reaction in cathodes. Early studies of cathode materials focused on different intercalated anions, such as hexafluorophosphate (PF_6_^−^), bis(trifluoromethanesulfonyl) imide (TFSI^−^), tetrachloroaluminate (AlCl_4_^−^), fluorosulfonyl(trifluoromethanesulfonyl) imide (FTFSI^−^), perchlorate (ClO_4_^−^), tetrafluoroborate (BF_4_^−^), bis(fluorosulfonyl) imide (FSI^−^), bis(perfluoroethylsulfonyl) imide (BETI^−^), tris(pentafluoroethyl) trifluorophosphate [(C_2_F_5_)_3_PF_3_^−^], trifluoromethanesulfonic (CF_3_SO_3_^−^), tetrafluoroaluminate (AlF_4_^−^) and difluoro (oxalate) borate (DFOB^−^) [72–75]. However, the relatively large radius of these anions leads to unsatisfactory intercalation capacity and structural disintegrations. In addition, solvent molecules are also likely to co-intercalate into the cathode materials during the anion intercalation process, thus accelerating the structural collapse and capacity loss [76]. To improve the performance of DIBs, it is necessary to modify and optimize the structure or composition of cathode materials. Considerable efforts, such as interlayer engineering, reinforcing materials with functional interfaces, designing nanostructured materials, and incorporating inorganic materials on the carbonaceous materials have been widely explored to enhance the electrochemical kinetics of cathode materials [77–79]. Interlayer engineering strategy can expand the interlamellar spacing, offer abundant electrochemical active sites and promote ion diffusion. Besides, synergetic effects between incorporated species and host materials bring in much more enhanced conductivity and surface modification. As a result, cathode electrodes with advanced interlayer design demonstrate a great enhancement of specific capacity/capacitance and rate performance [80]. For instance, Li et al. reported MoS_2_ with large layered spacing (~ 6.15 Å) as the cathode to store anion in Li-based DIBs [81]. Such a structure with enlarged interlayer distance effectively improves the anion intercalation kinetics and achieves great stability in a wide voltage window. Based on 1 M lithium difluoro(oxalato)borate (LiDFOB) electrolyte, dissociated DFOB^−^ ions can reversibly intercalate into MoS_2_ layers (Fig. 4a). The superior stability during the charge–discharge process is further investigated by the in-situ Raman test (Fig. 4b). Two typical peaks at 383 cm^−2^ (E_2g_) and 408 cm^−2^ (A_1g_) that originated from inherent in-plane and vertical-plane vibrations are observed for pristine MoS_2_ cathode. As the voltage increase, the A_1g_ peak shifts to higher wavenumber values, indicating the formation of MoS_2_ intercalation compounds, while the E_2g_ peak is almost no change, but the intensity decreases gradually with the charging, indicating the decrement of horizontal motions of Mo and S atoms. During the discharging process, the A_1g_ peak can recover back to its original state and the intensity of the E_2g_ peak increases gradually, further indicating the excellent reversibility of MoS_2_ with enlarged interlayers. A high specific capacity of 135 mAh g^−1^ has been achieved over 50 cycles for MoS_2_||graphite DIBs. Xu et al. developed a bilayer-structured V_6_O_13_ as cathode material for Li storage [82]. The lattice water forms layer hydroxyls and induces expanded interlayer spacing, which enables fast Li^+^ ions mobility and offers abundant binding sites, thus leading to the boosted Li storage performance.

Constructing advanced artificial SEI on cathode materials is another promising solution to enhance the structural integrity of cathode materials [83, 84]. The formation of a solid SEI usually inhibits further reduction reactions and enables them to maintain long-term cycling stability [85]. Li et al. controllably constructed a highly effective protector of the SEI layer on the graphite cathode (Fig. 4c) [86]. Such SEI significantly improves the structural stability and protects the graphite from the anion salvation effect and the deposition derived from electrolyte decomposition. The additional semicircle with smaller resistance at the relatively high frequency of the electrochemical impedance spectroscopy (EIS) profile indicates the SEI formation (Fig. 4d). These guarantee the stabilization of the graphite surface region. Therefore, the SEI-modified graphite (SMG) delivers a specific capacity of 84.5 mAh g^−1^ as coupling with Li anode, while the capacity of unmodified graphite (UMG) decreases to 75.1 mAh g^−1^. More importantly, SMG exhibits greatly promoted cyclic stability (98% capacity retention after 2000 cycles) than UMG (only 180 cycles), indicating the importance of surface modification in promoting the cycling performance of cell configuration (Fig. 4e). Designing nanostructural cathode materials with well-designed architectures have many structural advantages for electrochemical energy storage applications. The nanosized building blocks and large surface areas can offer a low diffusion energy barrier for the migration of electrons/ions, and thus can promote the reaction kinetics and allow full utilization of the active materials. Well-designed configurations with controlled size also help to provide facile stress relaxation, thus maintaining the integrity of the electrode and achieving a stable cycle lifespan. Li et al. proposed CuO hollow nanocube (HNC) cathode materials, which endow CuO with a high surface-to-volume ratio and interior voids as a physical buffer to accommodate the volume change [87]. The resulting particles have cubic shapes with average sizes of 60 nm (Fig. 4f). *Ex-situ* XRD results indicate that the CuO nanoparticles undergo a phase transformation upon contact with the electrolyte (Fig. 4g). Based on SO_2_-in-salt electrolyte, the Li||CuO HNC DIB delivers a high reversible capacity of 262.2 mAh g^−1^ with stable cycle performance up to 150 cycles (Fig. 4h). Although designing nanostructural materials can greatly improve the cathode electrochemical performance, the high surface areas of the electrode may lead to some side reactions. It should also be noted that the nanostructures will decrease the volumetric energy density of the electrodes. Fine-tuning the nanosized building blocks may address these issues. Recently, there is growing discussion of coating inorganic materials on the carbonaceous materials to restrain the successive decomposition of SEI and electrolyte, thereby keeping the structural integrity of the cathode [79, 88, 89]. For instance, Wu et al. stabilized the interfacial stability of the graphite cathode by applying a rigid/inert surface coating [90]. A thin amorphous Al_2_O_3_ layer was deposited on the graphite. This protective Al_2_O_3_ layer can serve as a stable artificial SEI and hinder the deposition of electrolyte decomposition products. Thus, the resultant Li-graphite DIBs achieve a long cycle life (80% capacity retention after 2700 cycles at 200 mA g^−1^).

### Electrolyte Optimization Strategies for DIBs

**Fig. 5:**
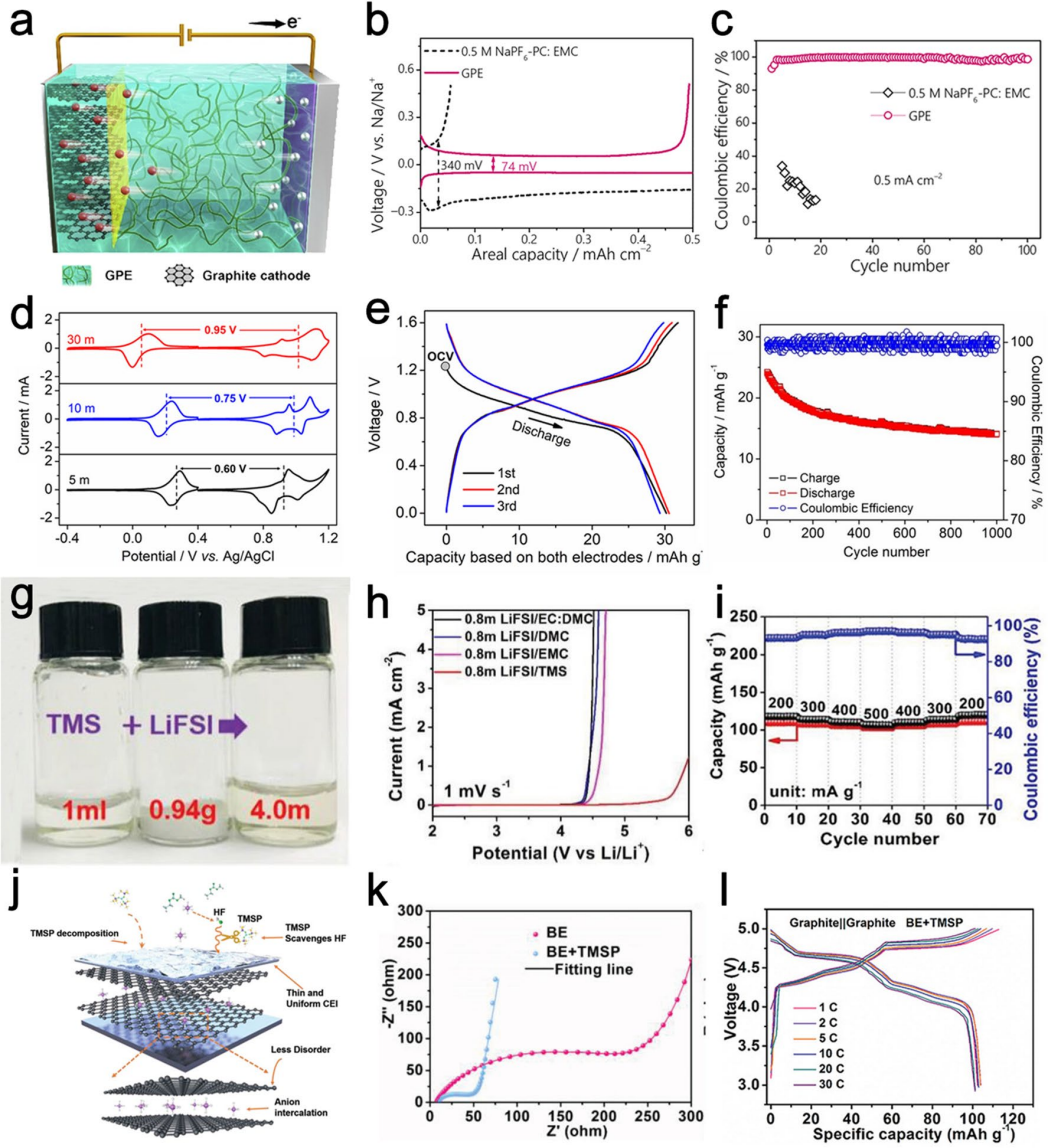
**a** Schematic illustration of the DIBs using GPE. **b** GCD profiles of Na||Cu cell using 0.5 M NaPF_6_-PC:EMC and GPE. **c** CE of Na plating-stripping in Na|0.5 M NaPF_6_-PC:EMC|Cu and Na|GPE|Cu cells. Panels (**a–c**) reproduced with permission from Ref. [99]. Copyright 2020, Elsevier. **d** CV curves tested in different ZnCl_2_ concentration electrolytes. **e** GCD curves and **f** cycling performance of Zn_3_[Fe(CN)_6_]_2_||Fc/C cell in 30 M ZnCl_2_ electrolytes. Panels (**d–f**) reproduced with permission from Ref. [103]. Copyright 2019, American Chemical Society. **g** Photographs of TMS, LiFSI, and 4.0 M LiFSI in TMS. **h** LSV plots under different electrolytes and **f** rate performance of Li||graphite DIB. Panels (**g–i**) reproduced with permission from Ref. [109]. Copyright 2018, John Wiley & Sons, Inc. **j** Schematic illustration of the unique functions of TMSP additive. **k** Nyquist plots of the Li||graphite cell with different electrolytes. **l** GCD curves of graphite||graphite in BE + TMSP. Panels (**j–l**) reproduced with permission from Ref. [111]. Copyright 2022, John Wiley & Sons, Inc

In DIBs, the electrolyte system not only serves as the ion transport medium but also acts as the active materials to provide both the cations and anions to be stored in the electrode [91, 92]. As a result, the effects of separate electrolyte components (salts, solvents, and additives), their oxidation/reduction at the cathode/anode sides, the “solvation effects” of anions, the formation and instability of SEI layer, the co-intercalation of solvent molecules into cathode materials are key issues to be addressed [93]. Besides, the cost and safety are also need to be considered, thus calling for more requirements apart from the characteristics of LIBs electrolyte systems [94]. The past decade has seen tremendous growth in the design and preparation of DIBs electrolytes with varying optimization strategies, including quasi-solid-state electrolytes (QSSEs), high-concentration electrolytes, high-voltage electrolytes, and the exploitation of electrolyte additives tailored for DIBs [95-97]. Development of QSSEs with excellent electrochemical stability is a possible means to improve their properties [98]. For instance, Xu et al. reported a multifunctional gel polymer electrolyte (GPE), which was in-situ synthesized by thermally polymerizing an ethoxylated pentaerythritol tetraacrylate (EPTA) monomer in an optimized liquid electrolyte with fluoroethylene carbonate (FEC) as co-solvent and 1,3-propanesultone (PS) as an additive (Fig. 5a) [99]. The crosslinked EPTA polymeric network not only keeps the GPE in a quasi-solid state to avoid electrolyte leakage but also effectively regulates cation and anion fluxes, which is favorable for anion intercalation into graphite and homogeneous Na plating, respectively. Meanwhile, the FEC and PS components significantly extend the electrolyte electrochemical window and promote the formation of low-resistance SEI on the Na anode, protecting against dendrite growth. As a result, the Na|GPE|Cu cell shows a much lower voltage hysteresis (~ 74 mV) between the Na plating and stripping plateaus than that of the Na|0.5 M NaPF_6_-propylene carbonate (PC):ethyl methyl carbonate (EMC)|Cu (~ 340 mV), indicating a low polarization of the GPE-based cell (Fig. 5b). Furthermore, the CE of Na|GPE|Cu cell attains 98.7% after 100 cycles, which is superior to the liquid electrolyte samples (Fig. 5c). When used in Na-based DIBs, the as-developed DIB demonstrates a high energy density (484 Wh g^−1^) with excellent long-term cycling performances (1000 cycles), which could be applied for low-cost energy storage.

Introducing high-concentration electrolytes is beneficial to achieve high intercalation capacity [100, 101]. Because of the reduced free solvent molecules, the co-intercalation phenomenon of solvent molecules at the cathode side can be greatly decreased, leading to enhanced CE and cycling stability of DIBs [102]. Wu et al. developed a concentrated 30 M ZnCl_2_ “water-in-salt” electrolyte, which can minimize the dissolution of the ferrocene anode, raise the potential of the cation cathode, and depress the potential of the anion anode, thus widening the full cell’s voltage [103]. The potential gap between the Zn_3_[Fe(CN)_6_]_2_ and the ferrocene/activated carbon (Fc/C) electrodes widens from 0.60 to 0.95 V from 5 to 30 M ZnCl_2_ electrolytes (Fig. 5d). As a result, the Fc/C||Zn_3_[Fe(CN)_6_]_2_ DIB exhibits a reversible capacity of 30 mAh g^−1^ (based on both the electrode materials) (Fig. 5e) and cycling life over 1000 times (Fig. 5f), much better than Fc/C||Zn_3_[Fe(CN)_6_]_2_ DIB with dilute ZnCl_2_ electrolyte. Xiang and co-workers reported a 7.5 M LiFSI in a carbonate electrolyte system [104]. Compared with diluted electrolytes, this highly concentrated electrolyte delivers enhanced intercalation capacity and cycling stability of the graphite cathode. Besides, the structural stability of the Al anode is also optimized with this highly concentrated electrolyte.

The anion intercalation process commonly occurs at high potential, thus developing high-voltage electrolytes is vital to heighten the specific capacity and lower the charge cutoff voltage to prevent electrolyte decomposition [105, 106]. Generally, traditional carbonate-based electrolyte usually possesses limited oxidation potential. Therefore, the exploration of high-voltage electrolyte systems beyond carbonates (sulfones, nitriles, phosphates, fluorinated carbonates, etc.) is urgently needed [107, 108]. Tong et al. reported an electrolyte system with 4 M LiFSI dissolved in tetramethylene sulfone (TMS) for DIBs (Fig. 5g) [109]. The as-prepared sulfone-based electrolyte shows much higher oxidative stability compared with carbonate electrolytes (Fig. 5h). A high oxidation potential of ~ 6.0 V is achieved and the gas formation under high working voltage is dramatically suppressed. The Li||graphite DIB constructed with such an electrolyte can deliver a capacity of 113.3 mAh g^−1^, along with good rate capability (Fig. 5i). Nevertheless, it is sometimes difficult to simultaneously satisfy the high oxidative and reductive stability within electrolytes composed of pure salts and solvents. Furthermore, there usually exist conflicts between the requirement for the electrolyte systems at the anode and cathode sides. To improve the charge/discharge efficiency and balance the electrolyte compatibility, introducing functional additives is an effective approach to address these problems [110]. Cheng et al. added tris(trimethyl-silyl) phosphite (TMSP) as an electrolyte additive to 3 M LiPF_6_ in EMC (Fig. 5j) [111]. The TMSP not only effectively inhibits the decomposition of electrolyte salts, but also produces a uniform and thin cathode electrolyte interphase (CEI) layer on graphite. This thin CEI layer improves the reaction kinetics and benefits the charge transfer (Fig. 5k). Hence, the rate capability and cycle performance induced by TMSP is greatly promoted compared with basic electrolytes (Fig. 5l).

## Practical Applications

LIBs with high energy density, no memory effect, and long cycle life, have dominated the market for a myriad of portable electronic devices [112, 113]. With the continuous development of technology and science, the progress of LIBs cannot adequately meet the surging demand for emerging energy applications [114]. More sustainable and high-performance energy storage systems are desired as future power sources. Among all available candidates, DIBs stand out as a promising energy storage device to replace state-of-the-art LIBs [115]. Hence, in the following section, we list some typical examples in these systems to highlight the advance of DIBs.

### Li/Na/K-based DIBs

**Fig. 6:**
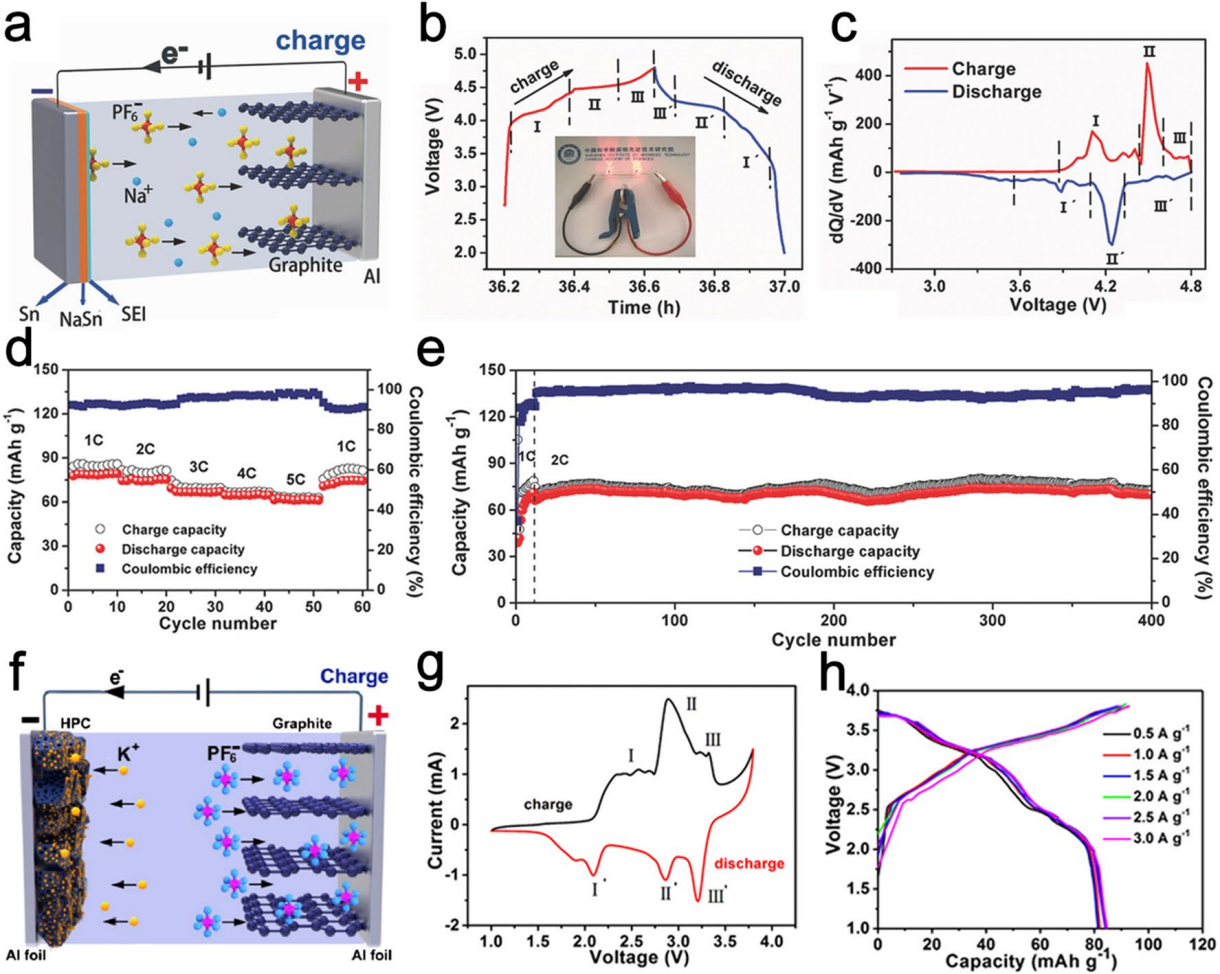
**a** Schematic illustration of the Na-based Sn||EG DIB cell. **b** GCD profile (inset is a photograph of a fully charged Sn||EG DIB to light up two LEDs in series), **c** CV curve, **d** rate capabilities, and **e** cycling performance of Sn||EG DIB. Panels (**a–e**) reproduced with permission from Ref. [121]. Copyright 2017, John Wiley & Sons, Inc. **f** Schematic illustration of the K-based HPC||EG DIB cell. **g** CV curves and **h** typical GCD curves of HPC||EG DIB. Panels (**f–h**) reproduced with permission from Ref. [122]. Copyright 2018, American Chemical Society

As a type of novel energy storage system, DIBs have attracted enormous interest due to their potential technological importance in achieving the possibility of energy storage with simultaneous high energy and high power density. There is a significant interest in finding advanced DIB systems for practical applications [116, 117]. For example, Han et al. reported the possibility of anion intercalation into Li_4_Ti_5_O_12_ (LTO) modified mesocarbon microbeads (MCMBs) by utilizing Li-containing electrolytes of 1 M LiPF_6_ dissolved in EMC/sulfolane (SL) (1:4 vol: vol) [118]. The LTO layer serves as a skeleton and provides electrocatalytic active sites to in-situ grow a compatible CEI layer. The synergetic LTO-CEI network can change the thermodynamic behavior during the PF_6_^−^ intercalation process and keep the structure from collapsing and electrolyte decomposition. A high energy density of ~ 200 Wh kg^−1^ is achieved based on LTO-modified MCMB||prelithiated MCMB full cell with 93.5% capacity retention after 1000 cycles. Recently, the concept of DIBs is successfully extended to Na/K-based reversible batteries. Developing lithium-free systems is a promising strategy for low-cost DIBs [119, 120]. Sheng et al. reported a Na-based DIB based on 1 M NaPF_6_ in ethylene carbonate (EC):DMC: EMC (1:1:1, v/v/v) as the electrolyte, using Sn foil as the anode, and EG as the cathode (Fig. 6a) [121]. This battery can work reversibly with high capacity over a high voltage window of 2.0–4.8 V. Three charge voltage regions between 4.0–4.58 V (stage I), 4.58–4.63 V (stage II), and 4.63–4.8 V (stage III), and three discharge voltage regions between 4.8–4.35 V (stage III’), 4.35–4.15 V (stage II’), and 4.15–3.6 V (stage I’) can be recognized, which correspond to different stages of PF_6_^−^ intercalation/deintercalation into graphite (Fig. 6b). The corresponding d*Q*/d*V* differential curves further prove each charge/discharge process stage (Fig. 6c). The Sn||EG DIB exhibits good Na storage performance in terms of high reversible capacity (78 mAh g^−1^ at 1C), good rate capability (61 mAh g^−1^ at 5C) (Fig. 6d), and enhanced cycling stability (94% capacity retention after 400 cycles) (Fig. 6e). Ding et al. proposed a K-based DIB by employing hierarchical porous carbon (HPC) as the anode and EG as the cathode (Fig. 6f) [122]. The different stages of PF_6_^−^ intercalation into graphite can be observed from the cyclic voltammetry (CV) curves (Fig. 6g). Owing to the hybrid mechanisms of the battery and capacitive reaction, the K-based DIB delivers a high capacity of 82 mAh g^−1^ at a high current density of 3 A g^−1^ with negligible capacity decay (Fig. 6h).

### Zn/Al-based DIBs

**Fig. 7:**
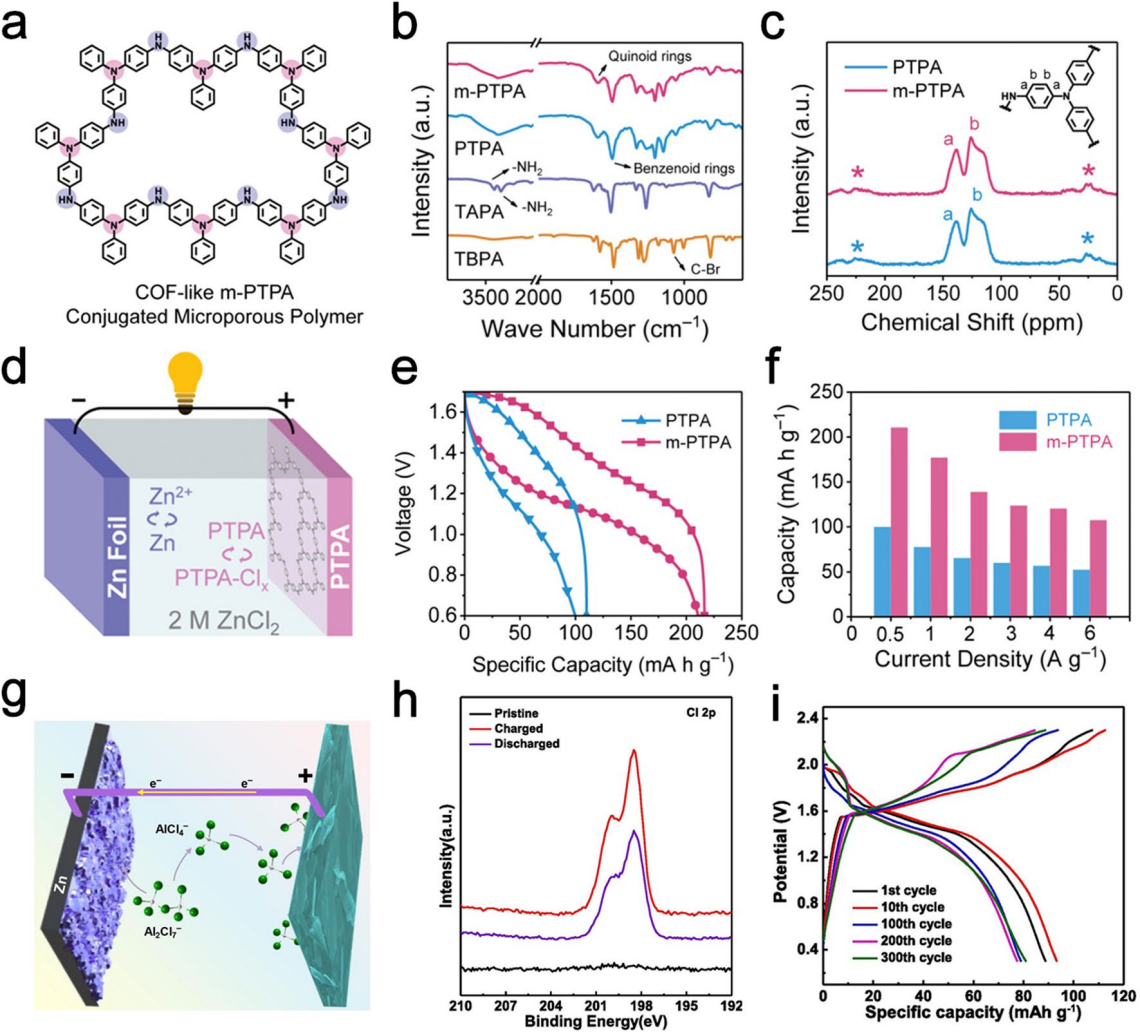
**a** Schematic illustration of the COF-like m-PTPA structure. **b** FTIR spectra of m-PTPA, PTPA and monomers. **c** Solid-state ^13^C NMR spectra of m-PTPA and PTPA. **d** Schematic illustration of the Zn||m-PTPA DIB cell. **e** GCD profiles and **f** rate capabilities of Zn DIBs consisting of m-PTPA and PTPA cathodes. Panels (**a–f**) reproduced with permission from Ref. [125]. Copyright 2021, John Wiley & Sons, Inc. **g** Schematic illustration of the Al-based Zn||graphite DIB cell. **h** XPS spectra of Al 2*p* of FG electrode before and after cycling. **i** GCD profiles of Zn||graphite DIB. Panels (**g–i**) reproduced with permission from Ref. [127]. Copyright 2022, Elsevier

Compared to Li/Na/K-ion storage, investigations on multivalent Zn/Al-ion-based DIBs are also investigated by different groups [123, 124]. Multivalent ions could transfer two or three electrons per ion during electrochemical reaction, holding the promise of a two- or three-fold increase in gravimetric capacities compared with monovalent DIBs. Moreover, multivalent ions (i.e., Mg^2+^, Zn^2+^, and Al^3+^) are commonly abundant element in the earth’s crust, and thus, providing new pathways towards economic energy storage solutions especially for large-scale stationary applications. For example, Zhang et al. reported a poriferous polytriphenylamine conjugated microporous polymer (m-PTPA) by coupling tris(4-aminophenyl)amine (TAPA) and tris(4-bromophenyl)amine (TBPA) units periodically [125]. The m-PTPA shows a covalent-organic-framework (COF)-like poriferous architecture consisting of rigid 3D conjugated networks, which is capable of accommodating Cl^−^ anions in a pseudocapacitive-dominated manner for energy storage (Fig. 7a). The Buchwald-Hartwig coupling is demonstrated by the disappearance of two typical signals belongs to the *v*_-NH2_ of TAPA (3340 and 3415 cm^−1^) and *v*_C-Br_ bonds of TBPA (1073 cm^−1^) and the emergence of benzenoid rings (*v*_C=C_, 1485 cm^−1^) and quinoid rings (*v*_C=C_, 1585 cm^−1^) in both nonporous conjugated PTPA and m-PTPA, signifying their polyaniline-like skeletons after the coupling reaction (Fig. 7b). Similarly, the two major peaks at 125 and 138 ppm observed from ^13^C nuclear magnetic resonance (NMR) spectra of PTPA and m-PTPA indicate the unsubstituted phenyl carbons and phenyl carbons connected to amines, respectively (Fig. 7c). The electrochemical performance of m-PTPA cathode is tested by assembling a Zn full cell in presence of a Zn foil anode and 2 M ZnCl_2_ aqueous electrolyte (Fig. 7d). Due to the rich amine functional groups distributed in the polymer skeleton, the m-PCMP displays increased capacities of 210.7 mAh g^−1^ at 0.5 A g^−1^ compared with nonporous conjugated PTPA (Fig. 7e). In addition, the m-PTPA electrode exhibits a high specific capacity of 107.5 mAh g^−1^ at 6 A g^−1^, indicative of its superior rate capability (Fig. 7f). Tu et al. discussed the possibility of Zn as the anode electrode of Al-based DIBs (Fig. 7g) [126]. The deposited Al particles on the Zn electrode are prior to Zn in the stripping reaction. As a result, the Zn electrode can keep structural integrality, thus presenting good corrosion resistance and cycling stability. X-ray photoelectron spectroscopy (XPS) characterizations of Cl 2*p* peaks confirm the successful insertion/de-insertion of Cl^−^ into graphite (Fig. 7h). The Zn-graphite full cell delivers an initial gravimetric capacity of 87.4 mAh g^−1^ when measured at 50 mA g^−1^ (Fig. 7i). Nevertheless, the diffusion nature of multivalent ions is still not well understood compared to monovalent Li ions. Sluggish solid-phase diffusion has been essential issues in developing intercalation cathode materials using multivalent ions. Therefore, find appropriate electrode materials is necessary to promote fast solid-phase diffusion of multivalent ions in DIBs systems.

## Summary and Perspectives

Energy and environmental issues are two inevitable problems in human development. Efficient and sustainable energy conversion and storage technologies are considered to be the most promising solutions to solve these problems [127]. DIBs possess outstanding characteristics such as high operating voltage, cost-effectiveness, and environmentally friendly that can be predicted to play an important role in large-scale energy storage devices [128]. This review outlines the recent developments and reaction mechanisms of DIBs. The optimization strategies involved in DIBs, including anode materials, cathode materials, and electrolyte systems are comprehensively discussed. So far, a wide range of DIBs modified by different strategies has been evaluated with varying electrochemical performance (Table 2). Their practical applications based on various DIB systems are also elaborated. Although the last decades have witnessed significant progress in DIBs devices, many challenges still exist and need to be tackled. Future research might be carried out based on the following aspects:

1. To further improve the overall performance of DIBs, individually optimizing and exploring innovative battery materials and electrolyte systems is still needed. Combining anode materials with conductive carbon can enhance the electronic conductivity and increase the transfer rate of cations in the anode. Nevertheless, the collapse and/or exfoliation of the carbon structure need to be assessed before modification. Incorporating functional groups between planar interlayers may help to stabilize the crystal structure. Designing nanostructured materials is expected to provide rich electrochemically active sites and large contact areas between electrode and electrolyte for mass diffusion and reactions. The small building blocks can reduce the diffusion length for fast ion/electron transport. However, the high surface areas may also lead to some severe side reactions between the electrode and electrolyte. Thus, understanding and suppressing these reactions need further consideration. Generally, the intercalation anions have a larger radius (i.e., 3.9 Å for TFSI^−^, 4.36 Å for PF_6_^−^, and 5.28 Å for TFSI^−^), which raises critical issues on sluggish ion diffusion kinetic as well as the insufficient electrochemical active sites that often result in poor specific capacities [129]. Therefore, more efforts should be devoted to further increasing the specific capacity of DIB cathodes. Besides, the high capacity and low working potential of the anode materials are essentially helpful for enhancing the energy density of DIBs. Of particular note is the efficient transportation of cations in anode materials and good compatibility with the electrolyte to match the anion intercalation kinetics in cathode. As mentioned, the electrolyte in DIBs plays a crucial role in the capacity and energy density, thus calling for more requirements apart from the characteristics claimed by LIBs. The construction of battery materials into hierarchically 3D morphologies, the preparation of small nanoclusters, the incorporation of carbonaceous material with high specific surface areas, and interlayer engineering are effective strategies to efficiently expose the active sites. Despite these developments, there still exist challenges for these rational designs. The hierarchical porosities and enlarged surface areas of electrode materials may need large amount of electrolyte to achieve a certain capacity and energy density, which will lead to high cost and low Coulombic efficiency. Hence, developing compatible low-cost electrolytes systems, such as non-lithium salts (i.e., NaPF_6_, KPF_6_, and Ca(PF_6_)_2_) with high solubility, are of great importance to reduce the overall cost. Besides, the lowered anion intercalation platform will improve the Coulombic efficiency even with traditional carbonate-based electrolytes.
2. The electrolyte in DIBs acts as active materials, and active anionic and cationic species and the amount of electrolyte should be adjusted according to the features of the host materials [130]. Generally, high-concentrated electrolyte leads to the reduction of electrolyte solvent, hence increasing the energy density of DIBs. Developing high-voltage electrolytes is another main research direction to improve the charge/discharge efficiency of DIBs. However, these electrolyte systems may easily lead to side reactions and severe corrosion of the current collects at the cathode side, especially under high working potential. Hence, introducing functional additives to promote the formation of stable SEI/CEI films is deserved to be investigated. Besides, developing a proper solid electrolyte that is stable is also essential for a long cycle life DIB. Unfortunately, the conventional electrolyte systems are generally inherited from LIBs, which are challenging to maintain the above characteristics at the same time, resulting in moderate energy density and limited cycle life. In order to optimize the electrolytes systems, it is of critical importance to uncover the effects of different electrolyte components (i.e., salts, solvents, and additives) on the performance of DIBs. Besides, the cost and safety are also important concerns that need to be considered for electrolyte design in practical use.
3. From the perspective of practical applications, rendering lower-cost and more efficient DIB configurations is surely one of the most important research directions in the future. Various scientific and technical challenges need to be tackled to bridge the gap between academic development and industrial manufacturing. Therefore, an in-depth understanding of the reaction processes, degradation mechanism, and side reactions under realistic operation conditions is of great importance to guide and improve the DIBs’ design. Currently, most of the reported characterization methods in DIBs are *ex-situ* techniques. The post-process nature limits the ability to study electrochemical processes of the materials, such as valance changes, surface, and interfacial reactions. Therefore, in-situ measurements and, in particular, operando measurements, such as in-situ/operando XRD, in-situ TEM, and in-situ X-ray absorption near-edge structure (XANES) are needed to monitor the structure evolution, the changes of the valance state and coordination environment, interfacial phenomena, and structural stability of electrodes in DIBs.

**Table 2:** Electrochemical performance of DIBs with different modification strategies

Towards	Strategy	DIBs configuration	Specific density	Cycling performance (cycles/capacity retention)	Rate capability	References
Anode	Compositing carbon	MoS_1.5_Te_0.5_@C||EG	218.6 mAh g^−1^ at 0.1 A g^−1^	1500/97%	101 mAh g^−1^ at 5 A g^−1^	[61]
		BP-C||graphite	82 mAh g^−1^ at 50 mA g^−1^	100/ca. 80.6%	–	[62]
	Porous structure	pK2TP||EG	68 mAh g^−1^ at 2C	2000/ca. 100%	45 mAh g^−1^ at 20C	[66]
		CuPcNA-CMP||graphite	245.3 mAh g^−1^ at 0.1 A g^−1^	500/ca. 89%	125.1 mAh g^−1^ at 5 A g^−1^	[67]
	Nanostructural crystalline	nAl@C||graphite	68 mAh g^−1^ at 2C	1000/94.6%	87 mAh g^−1^ at 20C	[70]
Cathode	Interlayer engineering	MoS_2_||graphite	90 mAh g^−1^	50/ca. 75%	–	[81]
	Artificial SEI	BiF_3_||NMO	ca. 100 mAh g^−1^ at 0.1 A g^−1^	40/ca. 50%	50 mAh g^−1^ at 1.6 A g^−1^	[85]
		Li||SMG	84.5 mAh g^−1^ at 0.2 A g^−1^	2000/98%	85 mAh g^−1^ at 0.3 A g^−1^	[86]
	Nanostructured materials	Li||CuO	208.8 mAh g^−1^ at 0.125 A g^−1^	100/79%	175 mAh g^−1^ at 0.25 A g^−1^	[87]
	Coating inorganic materials	Li||graphite	85 mAh g^−1^ at 0.1 A g^−1^	2700/80%	80 mAh g^−1^ at 2 A g^−1^	[90]
Electrolyte	Quasi-solid-state electrolytes	Al||graphite	103 mAh g^−1^ at 0.2 A g^−1^	2000/92%	82 mAh g^−1^ at 1 A g^−1^	[98]
		Na||graphite	86.3 mAh g^−1^ at 0.01 A g^−1^	100/86.6%	84.3 mAh g^−1^ at 0.5 A g^−1^	[99]
	High-concentration electrolytes	Fc||C	30 mAh g^−1^ at 0.03 A g^−1^	100/80%	–	[103]
		Al||graphite	89.8 mAh g^−1^ at 0.2 A g^−1^	1000/94.7%	68.1 mAh g^−1^ at 0.5 A g^−1^	[104]
	High-voltage electrolytes	Li||graphite	113.3 mAh g^−1^ at 0.2 A g^−1^	100/ca. 80.6%	103.6 mAh g^−1^ at 0.5 A g^−1^	[109]
	Electrolyte additives	Graphite||graphite	101.3 mAh g^−1^ at 0.1 A g^−1^	3000/96.8%	98.2 mAh g^−1^ at 2 A g^−1^	[111]

## References

[1] Liu T, Liu J, Li L, Yu L, Diao J (2022). Origin of structural degradation in Li-rich layered oxide cathode. Nature.

[2] Liu Y, Tao X, Wang Y, Jiang C, Ma C (2022). Self-assembled monolayers direct a LiF-rich interphase toward long-life lithium metal batteries. Science.

[3] Lemoine K, Hemon-Ribaud A, Leblanc M, Lhoste J, Tarascon J-M (2022). Fluorinated materials as positive electrodes for Li- and Na-Ion batteries. Chem. Rev..

[4] Li Y, Wu F, Li Y, Liu M, Feng X (2022). Ether-based electrolytes for sodium ion batteries. Chem. Soc. Rev..

[5] Zeng Y, Gordiichuk P, Ichihara T, Zhang G, Sandoz-Rosado E (2022). Irreversible synthesis of an ultrastrong two-dimensional polymeric material. Nature.

[6] Lv H, Pan Q, Song Y, Liu X-X, Liu T (2020). A Review on Nano-/microstructured materials constructed by electrochemical technologies for supercapacitors. Nano-Micro Lett..

[7] Lv C, Zhou X, Zhong L, Yan C, Srinivasan M (2022). Machine learning: an advanced platform for materials development and state prediction in lithium-ion batteries. Adv. Mater..

[8] Shan Y, Li Y, Pang H (2020). Applications of tin sulfide-based materials in lithium-ion batteries and sodium-ion batteries. Adv. Funct. Mater..

[9] Wu H, Liu Q, Guo S (2014). Composites of Graphene and LiFePO_4_ as cathode materials for lithium-ion battery: a mini-review. Nano-Micro Lett..

[10] Fang R, Chen K, Yin L, Sun Z, Li F (2019). The regulating role of carbon nanotubes and graphene in lithium-ion and lithium-sulfur batteries. Adv. Mater..

[11] Liang H, Wang L, Wang A, Song Y, Wu Y (2023). Tailoring practically accessible polymer/inorganic composite electrolytes for all-solid-state lithium metal batteries: a review. Nano-Micro Lett..

[12] Li M, Liu T, Bi X, Chen Z, Amine K (2020). Cationic and anionic redox in lithium-ion based batteries. Chem. Soc. Rev..

[13] Liu H, Lai W-H, Yang Q, Lei Y, Wu C (2021). Understanding sulfur redox mechanisms in different electrolytes for room-temperature Na–S batteries. Nano-Micro Lett..

[14] Chae S, Choi S-H, Kim N, Sung J, Cho J (2020). Integration of graphite and silicon anodes for the commercialization of high-energy lithium-ion batteries. Angew. Chem. Int. Ed..

[15] Rafique A, Ferreira I, Abbas G, Baptista AC (2023). Recent advances and challenges toward application of fibers and textiles in integrated photovoltaic energy storage devices. Nano-Micro Lett..

[16] Xia H, Zhang W, Cao S, Chen X (2022). A figure of merit for fast-charging Li-ion battery materials. ACS Nano.

[17] Ma D, Cao Z, Hu A (2014). Si-based anode materials for li-ion batteries: a mini review. Nano-Micro Lett..

[18] Kim H, Hong J, Park K-Y, Kim H, Kim S-W (2014). Aqueous rechargeable Li and Na ion batteries. Chem. Rev..

[19] Wu C, Tong X, Ai Y, Liu D-S, Yu P (2018). A review: enhanced anodes of Li/Na-Ion batteries based on yolk–shell structured nanomaterials. Nano-Micro Lett..

[20] Li H, Zhang W, Sun K, Guo J, Yuan K (2021). Manganese-based materials for rechargeable batteries beyond lithium-ion. Adv. Energy Mater..

[21] Ma W, Luo L-W, Dong P, Zheng P, Huang X (2021). Toward high-performance dihydrophenazine-based conjugated microporous polymer cathodes for dual-ion batteries through donor-acceptor structural design. Adv. Funct. Mater..

[22] Huang Y, Liang Z, Wang H (2020). A dual-ion battery has two sides: the effect of ion-pairs. Chem. Commun..

[23] Rüdorff W, Hofmann U (1938). Über Graphitsalze. Z. Anorg. Allg. Chem..

[24] F.P. McCullough, C.A. Levine, R.V. Snelgrove, Secondary battery, US Patent: US4830938, (1989)

[25] Carlin RT, Hugh C, Long D, Fuller J, Trulove PC (1994). dual intercalating molten electrolyte batteries. J. Electrochem. Soc..

[26] Guo Q, Kim K-I, Li S, Scida AM, Yu P (2021). Reversible insertion of I-Cl interhalogen in a graphite cathode for aqueous dual-ion batteries. ACS Energy Lett..

[27] Rodriguez-Perez IA, Zhang L, Wrogemann JM, Driscoll DM, Sushko ML (2020). Enabling natural graphite in high-voltage aqueous graphite||Zn metal dual-ion batteries. Adv. Energy Mater..

[28] Dai G, He Y, Niu Z, He P, Zhang C (2019). A dual- ion organic symmetric battery constructed from phenazine-based artificial bipolar molecules. Angew. Chem. Int. Ed..

[29] Yu H, Deng C, Yan H, Xia M, Zhang X (2021). Cu_3_(PO_4_)_2_: novel anion convertor for aqueous dual-ion battery. Nano-Micro Lett..

[30] Cui C, Wei Z, Xu J, Zhang Y, Liu S (2018). Three-dimensional carbon frameworks enabling MoS_2_ as anode for dual ion batteries with superior sodium storage properties. Energy Storage Mater..

[31] Li Y, Lu Y, Zhao C, Hu Y-S, Titirici M-M (2017). Recent advances of electrode materials for low-cost sodium-ion batteries towards practical application for grid energy storage. Energy Storage Mater..

[32] Placke T, Bieker P, Lux SF, Fromm O, Meyer H-W (2012). Dual-ion cells based on anion intercalation into graphite from ionic liquid-based electrolytes. Z. Phys. Chem..

[33] Zhang X, Tang Y, Zhang F, Lee C-S (2016). A novel aluminum-graphite dual-ion battery. Adv. Energy Mater..

[34] Yang CY, Chen J, Ji X, Pollard TP, Lu XJ (2019). Aqueous Li-ion battery enabled by halogen conversion-intercalation chemistry in graphite. Nature.

[35] Yu M, Sui Y, Sandstrom SK, Wu C-Y, Yang H (2022). Reversible copper cathode for nonaqueous dual-ion batteries. Angew. Chem. Int. Ed..

[36] Xu Q, Ding R, Shi W, Ying D, Huang Y (2019). Perovskite KNi_0.1_Co_0.9_F_3_ as a pseudocapacitive conversion anode for high-performance nonaqueous Li-ion capacitors and dual-ion batteries. J. Mater. Chem. A.

[37] Wu H, Ye Z, Zhu J, Luo S, Li L (2022). High discharge capacity and ultra-fast-charging sodium dual-ion battery based on insoluble organic polymer anode and concentrated electrolyte. ACS Appl. Mater. Interfaces.

[38] Wei C, Gong D, Xie D, Tang Y (2021). The free-standing alloy strategy to improve the electrochemical performance of potassium-based dual-ion batteries. ACS Energy Lett..

[39] Wu H, Li L, Yuan W (2022). Nano-cubic alpha-Fe_2_O_3_ anode for Li^+^/Na^+^ based dual-ion full battery. Chem. Eng. J..

[40] Ma W, Luo L-W, Huang X, Dong P, Chen Y (2023). Dihydrophenazine-based conjugated microporous polymer cathodes with enhanced electronic and ionic conductivities for high-performance aluminum dual-ion batteries. Adv. Energy Mater..

[41] Lei H, Wang H, Cheng B, Zhang F, Liu X (2022). Anion-vacancy modified WSSe nanosheets on 3D cross-networked porous carbon skeleton for non-aqueous sodium-based dual-ion storage. Small.

[42] Wu H, Hu T, Chang S, Li L, Yuan W (2021). Sodium-based dual-ion battery based on the organic anode and ionic liquid electrolyte. ACS Appl. Mater. Interfaces.

[43] Fang Y, Zheng W, Hu T, Li L, Yuan W (2022). N-doped carbon-coated mixed-phase SnS-SnS_2_ anode with carbon nanofibers skeleton for improving dual-ion battery in concentrated electrolyte. Energy Technol..

[44] Wang M, Tang Y (2018). A review on the features and progress of dual-ion batteries. Adv. Energy Mater..

[45] Xu Y, Ruan J, Pang Y, Sun H, Liang C (2020). Homologous strategy to construct high-performance coupling electrodes for advanced potassium-ion hybrid capacitors. Nano-Micro Lett..

[46] Sui Y, Liu C, Masse RC, Neale ZG, Atif M (2020). Dual-ion batteries: the emerging alternative rechargeable batteries. Energy Storage Mater..

[47] Li J, Fleetwood J, Hawley WB, Kays W (2022). From materials to cell: state-of-the-art and prospective technologies for lithium-ion battery electrode processing. Chem. Rev..

[48] Obrovac MN, Chevrier VL (2014). Alloy negative electrodes for Li-ion batteries. Chem. Rev..

[49] Ming F, Liang H, Huang G, Bayhan Z, Alshareef HN (2021). MXenes for rechargeable batteries beyond the lithium-ion. Adv. Mater..

[50] Lyu Y, Wu X, Wang K, Feng Z, Cheng T (2021). An overview on the advances of LiCoO_2_ cathodes for lithium-ion batteries. Adv. Energy Mater..

[51] Ji B, Zhang F, Song X, Tang Y (2017). A novel potassium-ion-based dual-ion battery. Adv. Mater..

[52] Jiang C, Xiang L, Miao S, Shi L, Xie D (2020). Flexible interface design for stress regulation of a silicon anode toward highly stable dual-ion batteries. Adv. Mater..

[53] Kwak K-H, Suh HJ, Kim A, Park S, Song J (2019). Reversible dual-ion battery via mesoporous Cu_2_O cathode in SO_2_-in-salt non-flammable electrolyte. Nano Energy.

[54] Guo Q, Kim K-I, Jiang H, Zhang L, Zhang C (2020). A high-potential anion-insertion carbon cathode for aqueous zinc dual-ion battery. Adv. Funct. Mater..

[55] Hu Z, Liu Q, Zhang K, Zhou L, Li L (2018). All carbon dual ion batteries. ACS Appl. Mater. Interfaces.

[56] Speer ME, Kolek M, Jassoy JJ, Heine J, Winter M (2015). Thianthrene-functionalized polynorbornenes as high-voltage materials for organic cathode-based dual-ion batteries. Chem. Commun..

[57] Sun Z, Zhu K, Liu P, Chen X, Li H (2022). Fluorination treatment of conjugated protonated polyanilines for high-performance sodium dual-ion batteries. Angew. Chem. Int. Ed..

[58] Wu D, Wang F, Yang H, Xu Y, Zhuang Y (2022). Realizing rapid electrochemical kinetics of Mg^2+^ in Ti-Nb oxides through a Li^+^ intercalation activated strategy toward extremely fast charge/discharge dual-ion batteries. Energy Storage Mater..

[59] Zhang Y, An Y, Yin B, Jiang J, Dong S (2019). A novel aqueous ammonium dual-ion battery based on organic polymers. J. Mater. Chem. A.

[60] Zhu K, Wu T, Sun S, van den Bergh W, Stefik M (2020). Synergistic H^+^/Zn^2+^ dual ion insertion mechanism in high-capacity and ultra-stable hydrated VO_2_ cathode for aqueous Zn-ion batteries. Energy Storage Mater..

[61] Liu Y, Hu X, Li J, Zhong G, Yuan J (2022). Carbon-coated MoS_1.5_Te_0.5_ nanocables for efficient sodium-ion storage in non-aqueous dual-ion batteries. Nat. Commun..

[62] Wrogemann JM, Haneke L, Ramireddy T, Frerichs JE, Sultana I (2022). Advanced dual-ion batteries with high-capacity negative electrodes incorporating black phosphorus. Adv. Sci..

[63] Zhang S, Wang M, Zhou Z, Tang Y (2017). Multifunctional electrode design consisting of 3D porous separator modulated with patterned anode for high-performance dual-ion batteries. Adv. Funct. Mater..

[64] Song C, Li Y, Li H, He T, Guan Q (2019). A novel flexible fiber-shaped dual-ion battery with high energy density based on omnidirectional porous Al wire anode. Nano Energy.

[65] Liu XX, Chen C, Blackwood DJ, Li NW, He Q (2022). Self-supported transition metal-based nanoarrays for efficient energy storage. Chem. Rec..

[66] Yu A, Pan Q, Zhang M, Xie D, Tang Y (2020). Fast rate and long life potassium-ion based dual-ion battery through 3D porous organic negative electrode. Adv. Funct. Mater..

[67] Wang H-G, Li Q, Wu Q, Si Z, Lv X (2021). Conjugated microporous polymers with bipolar and double redox-active centers for high-performance dual-ion, organic symmetric battery. Adv. Energy Mater..

[68] Liu B, Liu Y, Hu X, Zhong G, Li J (2021). N-doped carbon modifying MoSSe nanosheets on hollow cubic carbon for high-performance anodes of sodium-based dual-ion batteries. Adv. Funct. Mater..

[69] Liu Y, Deng W, Meng Z, Wong W-Y (2020). A tetrakis(terpyridine) ligand-based cobalt(ii) complex nanosheet as a stable dual-ion battery cathode material. Small.

[70] Tong X, Zhang F, Chen G, Liu X, Gu L (2018). Core-shell aluminum@carbon nanospheres for dual-ion batteries with excellent cycling performance under high rates. Adv. Energy Mater..

[71] Salunkhe TT, Kadam AN, Kidanu WG, Lee S-W, Nguyen TL (2021). A diffusion encouraged core-shell heterostructured Co_3_Sn_2_@SnO_2_ anode towards emerging dual ion batteries with high energy density. J. Mater. Chem. A.

[72] Fan L, Liu Q, Xu Z, Lu B (2017). An organic cathode for potassium dual-ion full battery. ACS Energy Lett..

[73] Li Q, Ma K, Hong C, Yang Z, Qi C (2021). High-voltage K/Zn dual-ion battery with 100,000-cycles life using zero-strain ZnHCF cathode. Energy Storage Mater..

[74] Kim K-I, Tang L, Mirabedini P, Yokoi A, Muratli JM (2022). [LiCl_2_]^-^ Superhalide: a new charge carrier for graphite cathode of dual-ion batteries. Adv. Funct. Mater..

[75] Li C, Yang H, Xie J, Wang K, Li J (2020). Ferrocene-based mixed-valence metal-organic framework as an efficient and stable cathode for lithium-ion-based dual-ion battery. ACS Appl. Mater. Interfaces.

[76] Rodriguez-Perez IA, Ji X (2017). Anion hosting cathodes in dual-ion batteries. ACS Energy Lett..

[77] Chen C, Li NW, Zhang XY, Zhang CH, Qiu J (2022). Interlayer-expanded titanate hierarchical hollow spheres embedded in carbon nanofibers for enhanced Na storage. Small.

[78] Stolz LS, Hochstädt SH, Röser SR, Hansen MRH, Winter MW (2022). Single-ion versus dual-ion conducting electrolytes: the relevance of concentration polarization in solid-state batteries. ACS Appl. Mater. Interfaces.

[79] Zhang M, Pei Y, Liu W, Liang R, Deng Y-P (2021). Rational design of interlayer binding towards highly reversible anion intercalation cathode for dual ion batteries. Nano Energy.

[80] Chen C, Li N-W, Wang B, Yuan S, Yu L (2020). Advanced pillared designs for two-dimensional materials in electrochemical energy storage. Nanoscale Adv..

[81] Li C, Lao B, Lia Z, Yin H, Yang Z (2020). Dual-ion battery with MoS_2_ cathode. Energy Storage Mater..

[82] Xu N, Ma X, Wang M, Qian T, Liang J (2016). Stationary full Li-ion batteries with interlayer-expanded V_6_O_13_ cathodes and lithiated graphite anodes. Electrochim. Acta.

[83] Zhang M, Shoaib M, Fei H, Wang T, Zhong J (2019). Hierarchically porous N-doped carbon fibers as a free-standing anode for high-capacity potassium-based dual-ion battery. Adv. Energy Mater..

[84] Zhang H, Xu D, Wang L, Ye Z, Chen B (2021). A polymer/graphene composite cathode with active carbonyls and secondary amine moieties for high-performance aqueous Zn-organic batteries involving dual-ion mechanism. Small.

[85] Zhang Z, Hu X, Zhou Y, Wang S, Yao L (2018). Aqueous rechargeable dual-ion battery based on fluoride ion and sodium ion electrochemistry. J. Mater. Chem. A.

[86] Li W-H, Ning Q-L, Xi X-T, Hou B-H, Guo J-Z (2019). Highly improved cycling stability of anion de-/intercalation in the graphite cathode for dual-ion batteries. Adv. Mater..

[87] Li S, Lee J-H, Hwang SM, Yoo J-B, Kim H (2021). Natural activation of CuO to CuCl_2_ as a cathode material for dual-ion lithium metal batteries. Energy Storage Mater..

[88] Han FC, Chen YX, Zhang JZ, Cai J, Xia XH (2021). Realizing ultralong-term cyclicability of 5 volt-cathode-material graphite flakes by uniformly comodified TiO_2_/carbon layer inducing stable cathode-electrolyte interphase. ACS Appl. Mater. Interfaces.

[89] Li WH, Li YM, Yang JL, Wu XL (2022). An integrated design of electrodes for flexible dual-ion batteries. Chemsuschem.

[90] Li WH, Li YM, Liu XF, Gu ZY, Liang HJ (2022). All-climate and ultrastable dual-ion batteries with long life achieved via synergistic enhancement of cathode and anode interfaces. Adv. Funct. Mater..

[91] Clarisza A, Bezabh HK, Jiang S-K, Huang C-J, Olbasa BW (2022). Highly concentrated salt electrolyte for a highly stable aqueous dual-ion zinc battery. ACS Appl. Mater. Interfaces.

[92] Hosaka T, Noda A, Kubota K, Chiguchi K, Matsuda Y (2022). Superconcentrated NaFSA-KFSA aqueous electrolytes for 2 V-class dual-ion batteries. ACS Appl. Mater. Interfaces.

[93] Jiang H, Han X, Du X, Chen Z, Lu C (2022). A PF_6_–permselective polymer electrolyte with anion solvation regulation enabling long-cycle dual-ion battery. Adv. Mater..

[94] Kim A, Jung H, Song J, Lee J, Jeong G (2021). Self-formulated Na-based dual-ion battery using nonflammable SO_2_-based inorganic liquid electrolyte. Small.

[95] Qin P, Wang M, Li N, Zhu H, Ding X (2017). Bubble-sheet-like interface design with an ultrastable solid electrolyte layer for high-performance dual-ion batteries. Adv. Mater..

[96] Wang Y, Zhang Y, Dong S, Zhou W, Lee P-K (2022). An all-fluorinated electrolyte toward high voltage and long cycle performance dual-ion batteries. Adv. Energy Mater..

[97] Wang Y, Zhang Y, Wang S, Dong S, Dang C (2021). Ultrafast charging and stable cycling dual-ion batteries enabled via an artificial cathode-electrolyte interface. Adv. Funct. Mater..

[98] Chen G, Zhang F, Zhou Z, Li J, Tang Y (2018). A flexible dual-ion battery based on PVDF-HFP-modified gel polymer electrolyte with excellent cycling performance and superior rate capability. Adv. Energy Mater..

[99] Xu X, Lin K, Zhou D, Liu Q, Qin X (2020). Quasi-solid-state dual-ion sodium metal batteries for low-cost energy storage. Chem.

[100] Kotronia A, Asfaw HD, Tai C-W, Hahlin M, Brandell D (2021). Nature of the Cathode-electrolyte interface in highly concentrated electrolytes used in graphite dual-ion batteries. ACS Appl. Mater. Interfaces.

[101] Li H, Kurihara T, Yang D, Watanabe M, Ishihara T (2021). A novel aqueous dual-ion battery using concentrated bisalt electrolyte. Energy Storage Mater..

[102] Li Z, Liu J, Niu B, Li J, Kang F (2018). A novel graphite-graphite dual ion battery using an AlCl_3_-[EMIm]Cl liquid electrolyte. Small.

[103] Wu X, Xu Y, Zhang C, Leonard DP, Markir A (2019). Reverse dual-ion battery via a ZnCl_2_ water-in-salt electrolyte. J. Am. Chem. Soc..

[104] Xiang L, Ou X, Wang X, Zhou Z, Li X (2020). Highly concentrated electrolyte towards enhanced energy density and cycling life of dual-ion battery. Angew. Chem. Int. Ed..

[105] Han X, Zhang H, Liu T, Du X, Xu G (2020). An interfacially self-reinforced polymer electrolyte enables long-cycle 5.35 V dual-ion batteries. J. Mater. Chem. A.

[106] Xiong Z, Guo P, Yang Y, Yuan S, Shang N (2022). A high-performance dual-ion battery-supercapacitor hybrid device based on LiCl in ion liquid dual-salt electrolyte. Adv. Energy Mater..

[107] Zafar ZA, Abbas G, Knizek K, Silhavik M, Kumar P (2022). Chaotropic anion based "water-in-salt" electrolyte realizes a high voltage Zn-graphite dual-ion battery. J. Mater. Chem. A.

[108] Zhang Y, Gui J, Li T, Chen Z, Cao S-A (2020). A novel Mg/Na hybrid battery based on Na_2_VTi(PO_4_)_3_ cathode: enlightening the Na-intercalation cathodes by a metallic Mg anode and a dual-ion Mg^2+^/Na^+^ electrolyte. Chem. Eng. J..

[109] Tong X, Ou X, Wu N, Wang H, Li J (2021). High oxidation potential approximate to 6.0 V of concentrated electrolyte toward high-performance dual-ion battery. Adv. Energy Mater..

[110] Ou X, Gong D, Han C, Liu Z, Tang Y (2021). Advances and prospects of dual-ion batteries. Adv. Energy Mater..

[111] Cheng Z, Guo L, Dong Q, Wang C, Yao Q (2022). Highly durable and ultrafast cycling of dual-ion batteries via in situ construction of cathode-electrolyte interphase. Adv. Energy Mater..

[112] Ferrari S, Falco M, Munoz-Garcia AB, Bonomo M, Brutti S (2021). Solid-state post Li metal ion batteries: a sustainable forthcoming reality?. Adv. Energy Mater..

[113] Fan E, Li L, Wang Z, Lin J, Huang Y (2020). Sustainable recycling technology for Li-ion batteries and beyond: challenges and future prospects. Chem. Rev..

[114] Jung S-K, Hwang I, Chang D, Park K-Y, Kim SJ (2020). Nanoscale phenomena in lithium-ion batteries. Chem. Rev..

[115] Li H, Okamoto NL, Hatakeyama T, Kumagai Y, Oba F (2018). Fast diffusion of multivalent ions facilitated by concerted interactions in dual-ion battery systems. Adv. Energy Mater..

[116] Kravchyk KV, Kovalenko MV (2019). Rechargeable dual-ion batteries with graphite as a cathode: key challenges and opportunities. Adv. Energy Mater..

[117] Gu J, Yuan Z, Wang H, Shen J, Ning J (2022). Local protonation of polyaniline induced by nitrogen-doped carbon skeleton towards ultra-stable Zn-organic batteries with a dual-ion insertion/extraction mechanism. Chem. Eng. J..

[118] Han X, Xu G, Zhang Z, Du X, Han P (2019). An in situ interface reinforcement strategy achieving long cycle performance of dual-ion batteries. Adv. Energy Mater..

[119] Xu Y, Feng J, Ma H, Zhu J, Zhang X (2022). Superior volumetric capability dual-ion batteries enabled by a microsize niobium tungsten oxide anode. Adv. Funct. Mater..

[120] Yan T, Ding R, Ying D, Huang Y, Huang Y (2019). An intercalation pseudocapacitance-driven perovskite NaNbO_3_ anode with superior kinetics and stability for advanced lithium-based dual-ion batteries. J. Mater. Chem. A.

[121] Sheng M, Zhang F, Ji B, Tong X, Tang Y (2017). A novel tin-graphite dual-ion battery based on sodium-ion electrolyte with high energy density. Adv. Energy Mater..

[122] Ding X, Zhang F, Ji B, Liu Y, Li J (2018). Potassium dual-ion hybrid batteries with ultrahigh rate performance and excellent cycling stability. ACS Appl. Mater. Interfaces.

[123] Kim K-I, Guo Q, Tang L, Zhu L, Pan C (2020). Reversible insertion of Mg-Cl superhalides in graphite as a cathode for aqueous dual-ion batteries. Angew. Chem. Int. Ed..

[124] Zhu Y, Yin J, Emwas A-H, Mohammed OF, Alshareef HN (2021). An aqueous Mg^2+^-based dual-ion battery with high power density. Adv. Funct. Mater..

[125] Zhang H, Zhong L, Xie J, Yang F, Liu X (2021). A COF-like N-rich conjugated microporous polytriphenylamine cathode with pseudocapacitive anion storage behavior for high-energy aqueous zinc dual-ion batteries. Adv. Mater..

[126] Tu J, Chang C, Wang M, Guan W, Jiao S (2022). Stable and low-voltage-hysteresis zinc negative electrode promoting aluminum dual-ion batteries. Chem. Eng. J..

[127] Wang H-G, Wang H, Si Z, Li Q, Wu Q (2019). A bipolar and self-polymerized phthalocyanine complex for fast and tunable energy storage in dual-ion batteries. Angew. Chem. Int. Ed..

[128] Yang K, Liu Q, Zheng Y, Yin H, Zhang S (2021). Locally ordered graphitized carbon cathodes for high-capacity dual-ion batteries. Angew. Chem. Int. Ed..

[129] Sun Z, Zhu K, Liu P, Li H, Jiao L (2021). Optimized cathode for high-energy sodium-ion based dual-ion full battery with fast kinetics. Adv. Funct. Mater..

[130] Chen C-Y, Matsumoto K, Kubota K, Hagiwara R, Xu Q (2020). An energy-dense solvent-free dual-ion battery. Adv. Funct. Mater..

